# Associations between Dietary Patterns, Fluoride Intake and Excretion in Women Exposed to Fluoridated Salt: A Preliminary Study

**DOI:** 10.3390/nu16193404

**Published:** 2024-10-08

**Authors:** Gina A. Castiblanco-Rubio, Michele Baston, Mauricio Hernandez-F, E. Angeles Martinez-Mier, Alejandra Cantoral

**Affiliations:** 1 Department of Dental Public Health and Dental Informatics, Indiana University School of Dentistry, Indianapolis, IN 46203, USA; ginacast@iu.edu (G.A.C.-R.); esmartin@iu.edu (E.A.M.-M.); 2 Health Department, Universidad Iberoamericana, Mexico City 01376, Mexico; 3 Research Institute for Equitable Development EQUIDE, Universidad Iberoamericana, Mexico City 01376, Mexico; mauricio.hernandez@ibero.mx

**Keywords:** fluorides, metabolism, biomarkers

## Abstract

Abundant information exists on fluoride intake and excretion in populations exposed to fluoridated water, but not fluoridated salt, where fluoride is eaten through a combination of foods and beverages. This study assessed associations between dietary patterns, fluoride intake and excretion in Mexican women exposed to fluoridated salt. We estimated dietary fluoride intake and excretion (mg/day) from 31 women using 24-h recalls (ASA24) and 24-h urine collections (HDMS diffusion method) and assessed agreement among both estimates of exposure with a Bland-Altman plot. Dietary patterns among the sample were explored by Principal Component Analysis and associations between these patterns and both fluoride intake and excretion were estimated. using Quantile Regressions. Median dietary fluoride intake and excretion were 0.95 and 0.90 mg/day, respectively, with better agreement at values below 1.5 mg/day. We identified three dietary patterns: “Urban Convenience”, “Plant-based” and “Egg-based”. The “Urban Convenience” pattern, characterized by dairy and convenience foods was associated with an increase of 0.25 mg and 0.34 mg of F in the 25th and 50th percentiles of intake respectively, (*p* < 0.01), and a marginal 0.22 mg decrease in urinary fluoride (*p* = 0.06). In conclusion, in this sample of Mexican women, a dietary pattern rich in dairy and convenience foods, was associated with both fluoride intake and excretion.

## 1. Introduction

The fluoride ion (F) is ubiquitous in the environment and constitutes a trace element of the diet [1]. Sustained exposure to adequate doses of F is associated with benefits for dental health, and communities around the globe have chosen to supplement water or salt with F to prevent and control dental caries [2]. Elevated intake of F is unequivocally associated with detrimental effects on human health, such as severe dental and skeletal fluorosis [3]. Emerging evidence suggests that side effects of fluoride (F) intake may be overlooked in populations exposed to levels currently deemed adequate for dental health [4,5]. This has warranted more research in F intake and exposure assessment in population groups.

Around 70% of the daily ingested F is excreted in the urine in a period of 24-h [6,7], and urinary F is the most widely used biomarker of exposure, both from dietary and non-dietary sources [8,9]. It is well documented that in groups of children and nonpregnant adults, urinary F correlates well with community water fluoride levels [10], with increasing urinary F levels as dietary intake increases [11]. However, less is known about the relationship between dietary intake of F and its urinary excretion in communities exposed to fluoridated salt.

The absorption of F from water in the gastrointestinal tract is a highly efficient process. Up to 100% of the ingested F from water can be absorbed by a fasting individual; however, the efficiency of this process can be greatly decreased if F is ingested with salt as a vehicle and concomitantly with other foods and beverages [12,13]. Additionally, sociodemographic confounders can impact an individual’s uptake of fluoride ions, including: age (as fluoride excretion increases with aging) [14], the types of foods consumed, dietary patterns [15], and cultural and geographic variations across different subpopulation groups. In fact, it has been reported that dietary patterns influence biomarker levels of several metals and trace elements [15]. Therefore, in groups of people exposed to salt fluoridation, where F is ingested through a variety of foods and beverages, the assumption that the excretion of F is directly proportional to estimated F intake (as it may apply to communities with fluoridated water), remains to be tested.

Dietary instruments such as 24-h recalls and Food Frequency Questionnaires, are useful tools to estimate usual intake of several nutrients, and for the identification of patterns of dietary intake in nutritional epidemiology [16]. On the other hand, F levels measured in 24-h urine collections are a biomarker of recent (contemporary) F exposure [9]. In order to understand how dietary intake relates to urinary excretion in a sub-population group exposed to fluoridated salt, we hypothesized that dietary F intake -as estimated with a 24-h dietary recall, is associated with 24-h urinary fluoride levels in a sample of women living in Mexico City—which has a salt fluoridation program implemented since 1981 [17]. Our aims were (1) To evaluate associations between dietary F intake and its urinary excretion; and (2) To identify dietary patterns within this sample and assess their association with both the intake and excretion of F.

## 2. Materials and Methods

### 2.1. Study Sample and Data Collection

**Figure 1:**
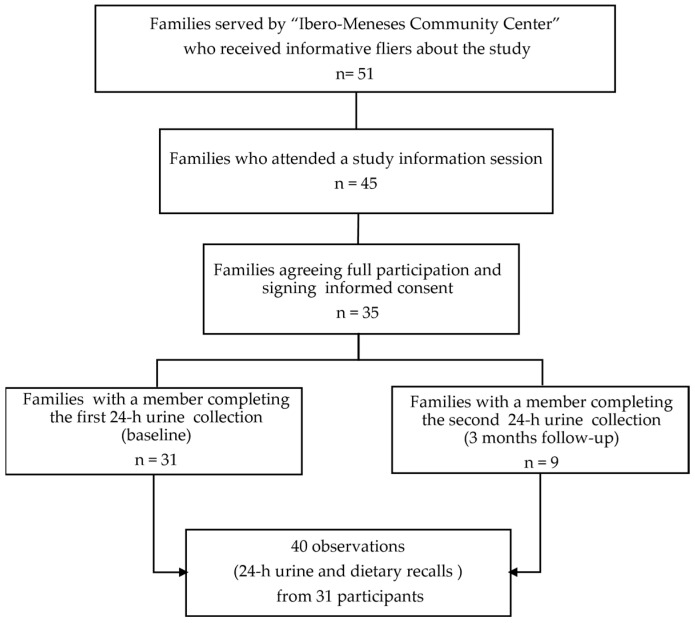
Flow chart of study recruitment strategy and final sample.

This fluoride study was nested in the parent study “Effect of community-based aromatic herb urban gardens on sodium intake and excretion”, which aimed at testing the effect of the implementation of community-based aromatic-herb urban gardens for the substitution/reduction of salt intake; with details published elsewhere [18]. The flowchart in Figure 1, depicts the process of recruitment and final study sample. Briefly, data collection was conducted from January 2022 to June 2022 in the Community Center “Centro Ibero-Meneses” in the neighborhood of Santa Fe, Mexico City. The community center is open to provide assistance to all community member, including individuals with medically managed metabolic disorders, including Type II Diabetes Mellitus, hypertension, and dyslipidemia, who do not have complications such as chronic kidney disease or require dialysis. Therefore, individuals with medical conditions were not excluded from participation. Of the 50 individuals who were invited to join, 35 agreed to participate and signed the informed consent or assent (in the case of minors). Ethical approval of the study was granted from the Ethics Commission and the Academic Council of Universidad Iberoamericana with the number 120/2021. All participants were women aged 14–65, with an average of 44 (±13 years); and 91% of them were primarily responsible for food preparation in the household. A total of 31 participants collected 24-h urine and dietary recalls at baseline, but only 9 were willing to provide urine samples and questionnaires 3 months later, after the intervention, for a total of 40 complete observations (dietary recalls + 24-h urine collections).

During the baseline visit, a general sociodemographic questionnaire, and anthropometric measurements (weight and height) were collected. Participants were provided with plastic containers, along with informative leaflets and instructional videos to guide them through the 24-h urine collection. On the day of the urine sample collection, participants were instructed to discard the first morning urine (immediately after getting up for the day) and register the time of the first urination as the start of the 24-h period. Urine collected over the day was pooled in a refrigerated plastic container, and the first urine of the following morning marked the end of the 24-h period.

To ensure that the dietary recall was concurrent with the 24-h urine sample, participants notified the research team of the completion of their urine collection through a text message and a trained nutritionist conducted the 24-h dietary recall over the phone. We used the 2020 ASA-24 version to capture detailed information on the foods and beverages consumed from 12:00 a.m. to 11:59 p.m. of the day the 24-h urine collection took place. The recall process lasted around 15–30 min.

To estimate the use of added salt, a trained research assistant visited the household of 21 participants, who reported to us the brand of salt and broth they regularly use for cooking. Participants were then asked for the brand, the amount and frequency at which they purchase salt and broth, and then we calculated the estimated daily amount of salt and broth ingested (in grams/day) by dividing the amount of salt or broth (e.g., 1000 g) by the frequency of purchase (e.g., 60 days), then divided by the number of people who regularly eat the food prepared at home.

### 2.2. Estimation of Dietary Fluoride Intake (Foods, Beverages and Added Salt)

We used The Automated Self-Administered 24-Hour Dietary Assessment Tool (ASA-24) [19]. The ASA 24 software uses the United States Department of Agriculture’s Food and Nutrient Database for Dietary Studies (USDA-FNDDS) to calculate nutrient intakes. First, we registered the project in the software, detailing every aspect, so we could then start applying the 24-h recall. The FNDDS does not include F values from neither foods or added salt and therefore, an output with fluoride intake estimates cannot be automatically obtained. To estimate F intake, we matched each food item reported by study participants with those available in a fluoride database that was specifically developed for the Mexican population, previously developed by our team and made publicly available elsewhere [20]. Briefly, the food groups analyzed for the construction of the database, had the following average fluoride content: (mean, µg F/100 g): Water: 11.85; Beverages: 23.23; Meat and Sausages: 191.47; Cereals and tubers: 77.81; Sweets, snacks and desserts: 83.97; Fruits: 3.78; Eggs: 2.32; Milk and Dairy: 27.18; Pasta and Soups: 41.23; Legumes: 84.91; Mexican Food: 67.85; and Vegetables: 19.85. In instances where specific foods were not present in the Mexican database, but were available in the USDA’s F database, the USDA value was imputed. The calculation of F intake from each food was performed by multiplying the amount of the food consumed (g) by the F content (µg/100 g) reported in the database, and then dividing the result by 100. The sum of the F content of each food item reported in the recall constitutes the fluoride intake per day (mg/day) from foods.

In some areas of Mexico, including Mexico City, fluoridated table salt is distributed for the prevention and control of dental caries (Official Mexican Norm NOM-040-SSA1-1993) [17]. To calculate F intake from added salt and/or broth (which can also be a source of fluoride intake), we used the estimations of daily added salt and broth (g) from the visits performed at the participant’s homes, as described above. Using our database of F content in salt and cubed/granulated broth distributed in Mexico City [21] (median F content: 142.5 µg F-/g), we matched the concentration in the salt or broth of the brands reported by the participants and multiplied it by the amount of daily added salt/broth estimated from direct observation. For participants with missing F estimates from salt (n = 9) the sample’s median of 0.25 mg F/day was imputed.

### 2.3. Determination of 24-h Urinary Fluoride Excretion and Urinary Creatinine

Urine samples were shipped to the Indiana University School of Dentistry’s Fluoride Research Lab. Urinary F was determined with the HMDS microdiffusion and fluoride-specific electrode method [22], as modified by Martinez-Mier et al. [23]. Each urine sample was thawed and vortexed at room temperature. A 1.0 mL aliquot was pipetted into a disposable petri dish. A trap of 0.05 N sodium hydroxide solution was placed in the form of five equal drops on the inside of the lid. After a tight sealing with petroleum jelly and burning a small hole into each lid, HMDS-saturated 3 N sulfuric acid was pipetted, and the F was allowed to diffuse overnight at room temperature. The following day, the petri dish was opened, and the solution of diffused F contained in the lid was recovered, buffered to pH 5.2 with acetic acid 0.1 M, and then diluted in deionized water. All analyses were performed in duplicates. The concentration of F was then measured using a fluoride ion selective electrode coupled to a pH/ISE meter (Orion™ Fluoride Electrode and Dual Star™ pH-meter, Thermo Scientific, Waltham, MA, USA). A calibration curve using fluoride standards (Orion™ ISE calibration standards) was constructed following the same procedure. Millivolt readings from the samples were recorded and the unknown concentrations of F from urine samples were determined using the equation that explained the relationship between the log of the F concentration of the standards and their corresponding millivolt readings (R^2^ > 0.9). Testing included a daily standard check using a F standard after sample analysis. Urinary creatinine in 24-h urine samples was determined using the Jaffe method (Cayman Chemical, Creatinine Colorimetric Assay Kit, 500701, Ann Arbor, MI, USA).

### 2.4. Statistical Analyses

We checked the normality of the distributions of both dietary F intake and excretion using the Shapiro–Wilk test. Skewed continuous variables were summarized using percentiles and interquartile ranges. The differences between visits 1 and 2 for continuous variables were evaluated with the Wilcoxon matched-pairs signed-rank test. We tested the correlation among dietary F intake and 24-h urinary fluoride excretion using the Spearman correlation test (non-parametric) and then verified agreement among both measurements using a Bland-Altman plot. After identifying a non-linear and negative (but concordant) relationship among dietary fluoride intake and urinary excretion, we explored dietary patterns among the sample, extracting data from the 24-h dietary recalls to perform a Principal Component Analysis (PCA). From the recalls, we identified 15 food groups. Four groups were excluded due to their minimal consumption within the sample (fast food, Mexican food, legumes, and seafood). We calculated the standardized values of the base-10 logarithms of fluoride intake and examined correlations among the food groups, following the procedure by Samuels [24]. All 11 food groups were retained for further analysis as all correlations were below 0.8. We then applied a non-rotating PCA to identify the optimal number of factors according to the criterion of achieving a cumulative variance proportion of at least 50%, which was found to be three food groups and no food group met the criterion for elimination based on communalities less than 0.2. Afterwards, we conducted a PCA with Varimax rotation, restricted to the three factors, and all food groups had load factors greater than 0.3. We saved the regression scores and estimated a linear regression with total fluoride consumption as the dependent variable and the factors as explanatory variables. The three identified factors accounted for 30.78% of the total variance of dietary F intake. Finally, to estimate the associations between dietary patterns and the dietary intake and excretion of F, we performed quantile regressions (suitable for variables with skewed distributions), at the 25, 50 and 75 percentiles. All statistical analyses were performed using STATA v17.0 (Stata Corp LP, College Station, TX, USA).

## 3. Results

### 3.1. Estimation of 24-h Dietary Fluoride Intake and Urinary Excretion

As expected, estimates of dietary intake of F had skewed distributions. Median dietary F intake for all 40 observations from the 31 participants was 0.95 mg F/day, with the estimates of salt and broth intake representing about 25% of the total estimated dietary intake. The median estimate for this sample is lower than the recommendation from the Institute of Medicine (OIM) of 3 mg F/day for females 14 and older. The median urinary excretion of F for all 40 observations was estimated at 0.9 mg F/day, with medians of 0.89 and 1.09 mg F/day for visits 1 and 2, respectively. Table 1 shows estimates stratified by visits. For visit one, 31 participants provided dietary recalls and 24-h urine samples, whereas only 9 out of the 31 provided a second dietary recall and a urine sample for visit two. Although median estimates from visit 2 were higher than those of visit 1, there were no statistically significant differences among the two visits.

### 3.2. Relationship between Dietary Fluoride Intake Estimated with the ASA-24 Dietary Recall and 24-h Urinary Fluoride Excretion

**Figure 2:**
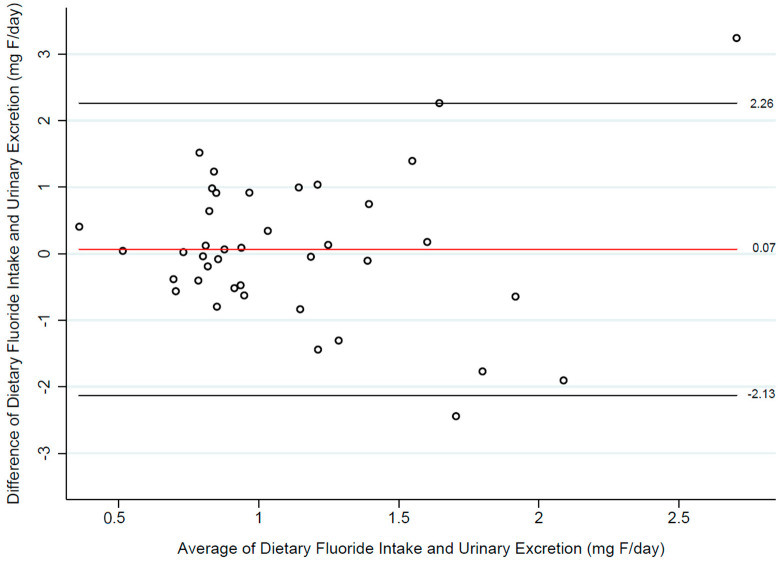
Agreement between estimates of dietary fluoride intake (ASA24 dietary recall) and 24-h urinary fluoride levels.

Exploratory statistics revealed that in the sample, the dietary intake of F and its urinary excretion did not follow a linear relationship, and both variables were found to be independent (Spearman’s rho: −0.25; *p* = 0.12). We therefore explored whether estimates of dietary F intake from the 24-h recall, and urinary F levels agreed with one another, using the procedure by Bland and Altman (Figure 2). Using this method, we found that dietary F intake levels estimated with the 24-h dietary recall may be 2.26 mg above or 2.13 mg below urinary F levels, with better agreement when F levels are at values below 1.5 mg/day.

### 3.3. Characterization of Identified Dietary Patterns

Using a data-driven exploratory method (Principal Component Analysis, PCA), we identified three dietary patterns based on the shared variance across the different foods reported within the sample.

Pattern 1 “Urban Convenience Diet”: This dietary pattern, is characterized by the consumption of beverages (water, coffee, soda, tea, milk, juice), cereals (tortilla, rice, bread, pasta), sweets, sweet bread, snacks, dairy products, and salsas. It reflects contemporary food choices and eating habits that are influenced by urbanization and changing lifestyles. It also acknowledges the access to diverse food options, including convenience foods, which are readily available. This consumption pattern is influenced by a combination of cultural traditions, urban lifestyles, and changing dietary preferences. While some aspects of this pattern align with the convenience and availability of urban environments, it’s important to note that variations exist within the population.

Pattern 2 “Plant-based diet”: This dietary pattern embodies the essence of a traditional Mexican diet, composed of fruit, soups and pasta, vegetables, and salsas (sauces). It brings together a variety of flavors that resonate with the country’s culinary heritage.

Pattern 3 “Egg-based pattern”: This pattern neither contributes significantly to fluoride consumption nor to its excretion, primarily due to the low fluoride content in eggs, being a prominent food group of this pattern.

### 3.4. Association between Dietary Patterns and 24-h Fluoride Intake and Excretion

**Figure 3:**
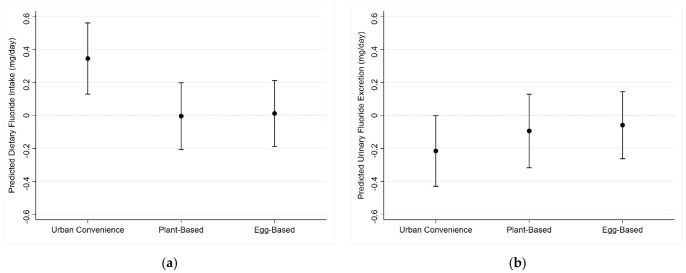
Effect of dietary patterns on the 50th percentile of dietary fluoride intake (95% Confidence Intervals: (**a**) Dietary Fluoride Intake; (**b**) Urinary Fluoride Excretion.

Using quantile regression, we found that the “Urban Convenience” dietary pattern was associated with an increase of 0.25 mg and 0.34 mg of F in the 25th and the 50th percentiles of dietary F intake (*p* < 0.01), respectively. On the other hand, the same dietary pattern was marginally associated (*p* = 0.06) with a 0.22 decrease in urinary fluoride excretion (Figure 3). We did not find associations among dietary patterns and dietary fluoride at any other quantiles of intake (Table 2).

## 4. Discussion

In our study, we found an association between a diet high in convenience foods and dairy (termed the “Urban Convenience Diet”) and the intake and excretion of dietary F in a sample of Mexican women. Our novel approach considers fluoride intake as part of dietary patterns, rather than in isolation, and questions the accuracy of crude dietary intake estimates in reflecting biologically relevant exposures. Interestingly, while the Urban Convenience Diet was positively associated with F intake, it was negatively associated with F excretion. This contradicts the assumption of a linear relationship between intake and excretion [6].

There are two potential explanations for our findings: (1) The Urban Convenience Diet may include foods that when eaten together with fluoride, inhibit its intestinal absorption, resulting in lower urinary excretion [12], or (2) The Urban Convenience Diet may promote the reabsorption of fluoride in the renal tubules, leading to a longer re-circulation time in the plasma before its excretion [14]. The first explanation is supported by our finding of a moderate, significant correlation between the Urban Convenience Pattern, and estimated dietary calcium intake (r = 0.37, *p* = 0.02). It is well-known that simultaneous intake of fluoride and calcium-rich foods reduces fluoride’s gastric and intestinal absorption; this is because fluoride and calcium react to form insoluble salts that are excreted in the feces [14]. The second explanation −higher fluoride reabsorption in the renal tubules, could be due to the overall acidity of the diet. Alkaline diets are associated with higher urinary excretion of fluoride, whereas acidic diets have been linked to lower urinary excretion due to increased recirculation of fluoride in the plasma [14,25]. The Urban Convenience pattern is characterized by the consumption of foods such as dairy, sodas and salsas, which are high in both salt (and therefore, F), but also in acid precursors. It is important to note that in this study, the dietary intake of fluoride was estimated from a dietary questionnaire, which like any dietary instrument, is subject to measurement error [16]. Although, we cannot definitively state the reasons behind the observed associations, we can say that the urinary excretion of fluoride may not necessarily follow a monotonic relationship with F intake, and that this relationship will depend on the combination of foods with which fluoride is ingested, and the overall eating pattern. For instance, there are individual foods with high fluoride levels (such as fast foods and sea food) [20], however it is the context of their ingestion the one determining the bioavailability of the fluoride ion. When using dietary questionnaires to estimate F intake in exposure assessment, it is crucial to understand that a higher estimated intake does not necessarily mean a higher biologically relevant (bioavailable) exposure to F and interpret the data within these limitations.

Dietary recalls and questionnaires are well-established tools for estimating dietary exposures in nutritional epidemiology [16] and are becoming a cost-effective alternative for assessing F exposure in environmental epidemiology studies [5,26]. However, our findings highlight that crude estimates do not account for factors affecting nutrient bioavailability, potentially increasing the risk of exposure misclassification. Using a Bland-Altman plot (Figure 2), we observed better agreement between fluoride intake estimates and urinary fluoride measurements at exposure levels below 1.5 mg/day. Beyond these levels, the likelihood of error increased. For fluoride exposure assessment, it is desirable to use instruments with higher precision also at high exposure levels, as these data points could provide valuable information on potential side effects. Our data suggests that the 24-h recall may not be suitable to substitute 24-h urinary fluoride measurements; however, this was a preliminary study, and a larger sample would be needed to confirm this finding.

This study had both strengths and limitations. Among its strengths, we collected 24-h urine samples and had a professional nutritionist administer the dietary recall. Our fluoride database was specifically developed for the population under study, and our study personnel visited the participants’ homes to make individual estimates of salt intake. On the other hand, limitations include that the current analyses were nested within another study that included a sodium intake reduction intervention [18], and that participants with metabolic disease were not excluded from participation. Although it is known that acid/base disorders can influence the excretion of fluoride [14], families served by Centro Meneses were under medical treatment, which decreases the likelihood of confounding. Lastly, the age range of the participating women was wide (while it is well known that the excretion of fluoride increases with age) [14]. To address these limitations, our quantile regression models controlled for each participant’s age and the study visit (1st or 2nd visit) at which the sample was collected. In an abundance of precaution, we also measured urinary creatinine and included it as a variable in our urinary excretion models to account for the effects of urine dilution.

## 5. Conclusions

We found that in this sample of Mexican women exposed to fluoridated salt, a dietary pattern characterized by dairy and convenience foods influences both the intake and excretion of fluoride. Under these conditions, the dietary intake of fluoride as estimated with a 24-h dietary recall and its 24-h urinary excretion were not linearly related and had better agreement at levels below 1.5 mg F/day.

## Figures and Tables

**Table 1:** Descriptive statistics of dietary fluoride intake as estimated from the 24-h dietary recalls, salt and broth estimations, and urinary fluoride excretion (both in mg/day).

	Visit 1 (n = 31)							Visit 2 (n = 9)					
	min	p25	p50	p75	max	IQR	min	p25	p50	p75	max	IQR	*p*-Value *
Dietary fluoride intake (mg/day)													
24-h dietary recall	0.17	0.41	0.66	1.06	2.52	0.65	0.20	0.34	0.83	1.44	4.07	1.09	0.43
Salt + broth	0.09	0.22	0.25	0.29	0.79	0.07	0.20	0.25	0.25	0.29	0.67	0.04	>0.9
Total (recall + salt + broth)	0.42	0.65	0.91	1.34	2.78	0.68	0.45	0.58	1.16	1.77	4.33	1.18	0.43
**Urinary fluoride (mg/day)**	0.03	0.51	0.89	1.26	3.04	0.75	0.82	0.99	1.09	1.25	2.93	0.26	0.36

p25, p50 and p75: 25th, 50th and 75th percentile, respectively. IQR: interquartile range. * Wilcoxon matched pair signed rank test.

**Table 2:** Quantile regression of the relationship between dietary patterns and dietary fluoride intake (Model 1) and urinary fluoride excretion (Model 2).

n = 40	0.25 Quantile		0.50 Quantile		0.75 Quantile	
	Estimate	*p*-Value	Estimate	*p*-Value	Estimate	*p*-Value
**Model 1: Dietary Fluoride Intake (mg/day)**						
Urban convenience	0.25	0.01	0.34	0.00	0.35	0.15
Plant-based	−0.08	0.35	0.00	0.97	0.06	0.80
Egg-based	−0.05	0.57	0.01	0.91	−0.03	0.90
**Model 2: Urinary fluoride excretion (mg/day)**						
Urban convenience	−0.20	0.10	−0.22	0.06	−0.13	0.60
Plant-based	0.04	0.75	−0.09	0.42	−0.25	0.33
Egg-based	−0.18	0.12	−0.06	0.57	−0.16	0.49

Model 1 (intake): adjusted for age, visit and total energy intake. Model 2 (excretion): adjusted for age, visit, fluoride from added salt and urinary creatinine.

## Data Availability

The raw data supporting the conclusions of this article will be made available by the authors on request.
